# Morphology engineering of basidiomycetes for improved laccase biosynthesis

**DOI:** 10.1007/s10529-015-2019-6

**Published:** 2015-12-23

**Authors:** Anna Antecka, Michał Blatkiewicz, Marcin Bizukojć, Stanisław Ledakowicz

**Affiliations:** Department of Bioprocess Engineering, Lodz University of Technology, ul. Wolczanska 213, 90-924 Lodz, Poland

**Keywords:** Basidiomycetes, Fungal morphology, Laccase, Microparticle-enhanced cultivation

## Abstract

**Objective:**

This work is the first application of a morphological engineering technique called microparticle-enhanced cultivation (MPEC) aimed at the facilitation of laccase production in the submerged cultures by two basidiomycetes species *Cerrena unicolor* and *Pleurotus sapidus*.

**Results:**

The positive effect of the applied 10 μm Al_2_O_3_ microparticles at concentrations from 5 to 30 g Al_2_O_3_ l^−1^ was shown. Laccase activity increased 3.5-fold for *C. unicolor* and 2-fold for *P. sapidus* at 15 g Al_2_O_3_ l^−1^ on 9 and 14 day of the cultivation, respectively, compared to the control culture without microparticles. The increase of laccase activity in the cultivation broths was caused by the action of Al_2_O_3_ microparticles on the agglomeration of hyphae. It led to the decrease of the size of the pellets, (on average by 2 mm for *C. unicolor*), the change of their shape (star-shaped pellets for *C. unicolor*) and the change of their structure (more compact pellets for *P. sapidus*).

**Conclusions:**

Application of MPEC for the submerged cultures of two laccase-producing basidiomycetes proved successful in increasing of enzyme production.

## Introduction

Laccase (EC 1.10.3.2) is a copper-containing oxidase that catalyze reduction of O_2_ to H_2_O using the variety of phenolic substrates as hydrogen donors. Laccase can convert o- and p-diphenols, aminophenols, polyphenols, polyamines and recalcitrant non-phenolic lignin compounds (Mayer and Staples 2002). Therefore, this enzyme can be applied for a number of biotechnological problems related to the degradation or chemical modification of structurally diverse compounds, being either xenobiotics or naturally occurring aromatic compounds (Couto and Herrera 2006; Imran et al. 2012).

However, the cultivation of fungal, especially basidiomycetes, cultures is a more complicated task than that of bacterial ones as the individual fungal strain can demonstrate a wide range of morphologies dependent on the age of mycelium, cultivation media composition, pH shifting and mechanical stress (Krull et al. 2013). What is more, fungal morphology, namely diameter of pellets and their structure, affects the titers of produced metabolites to the high extent (Bizukojc and Ledakowicz 2010) and changing fungal morphology with the use of morphological engineering techniques, like microparticle-enhanced cultivation (MPEC) often leads to improving the production of metabolites and enzymes (Krull et al. 2013).

In the pioneering study the use of such inorganic microparticles as Al_2_O_3_ or talc added to the fungal cultures, namely MPEC technique, was introduced to influence on *Caldariomyces fumago* morphology and enhance chloroperoxidase production (Kaup et al. 2008). Other authors also observed that intentional supplementation of microparticles to fungal cultures generally stimulated growth of these organisms as well as biosynthesis of selected enzymes. MPEC applied for *Aspergillus niger* led to the formation of freely dispersed mycelium, which occurred to be favorable for high productivity of glucoamylase (Driouch et al. 2010). Talc microparticles decreased pellet diameter in *Aspergillus terreus* and improved lovastatin production (Gonciarz and Bizukojc 2014).

The only literature data concerning the effect of microparticles on laccase production come from Tišma et al. (2012). They explored its production by *Trametes versicolor* on industrial waste and found that microparticles of CaCO_3_ unintentionally present in the waste substrate changed fungal morphology and enhanced laccase activity. Upon these findings we attempted to apply MPEC for the first time for basidiomycetous white rot fungi *Cerrena unicolor* and *Pleurotussapidus* in order to intensify laccase biosynthesis.

## Methods

*Cerrena unicolor* (Bull. ex Fr.) Murr. strain 137 used in this study was obtained from the culture collection of Department of Biochemistry, Maria Curie-Skłodowska University, Lublin, Poland. Stock cultures were maintained on malt extract agar (MEA) slants at 4 °C.

*Pleurotus sapidus* (DSM 8266) strain was obtained from DSMZ and maintained on malt extract peptone agar.

The experiments were run in 500 ml shake flasks containing 200 ml of Lindeberg–Holm liquid medium as described previously (Janusz et al. 2007) for *C. unicolor* and modified Zorn medium (Zorn et al. 2003) for *P. sapidus*. For *P. sapidus* the additional experiments were required to find the optimal Cu^2+^ concentration in the medium. Liquid media were sterilized at 121 °C and 1.6 bar for 15 min.

For the cultivations with microparticles the following concentrations of Al_2_O_3_ were selected: 5, 10, 15, 20 and 30 g Al_2_O_3_ l^−1^. The use of Al_2_O_3_ was justified by the fact that out of many types of microparticles used in MPEC they belong to the most inactive ones (Etschmann et al. 2015). The portions of microparticles were prepared separately and sterilized in the same conditions as the culture medium. In all experiments the inoculation was made with a 5 ml of homogenized fungal mycelium from the overgrown agar plates. Next, Al_2_O_3_ was added, excluding the flasks for the control run. All flasks were incubated on a rotary shaker (Sartorius, Germany) at 28 °C and constant rotary speed of 110 rpm for 14 days. The liquid medium was separated from biomass by filtration on paper filters (Filtrak, Germany). The supernatant was used for further analyses.

Laccase activity was determined in the culture liquid by measuring the oxidation of 0.5 mM syringaldazine dissolved in ethanol buffered in 0.1 M citrate-buffer (pH 5.6, ε_525_ = 65 mM^−1^ cm^−1^). All spectrophotometric measurements were carried out using a UV-300 spectrophotometer (Unicam, Cambridge, UK). Enzyme activities were expressed in U l^−1^, where units (U) were defined as 1 µmol of product formed per one minute. Biomass concentration was assayed as dry weight of the fungal mycelium. All chemicals were obtained from Sigma-Aldrich (Steinheim, Germany) and Merck (Darmstadt, Germany). Al_2_O_3_ microparticles of mean diameter equal to 10 µm were purchased from Sigma-Aldrich.

## Results and discussion

**Fig. 1:**
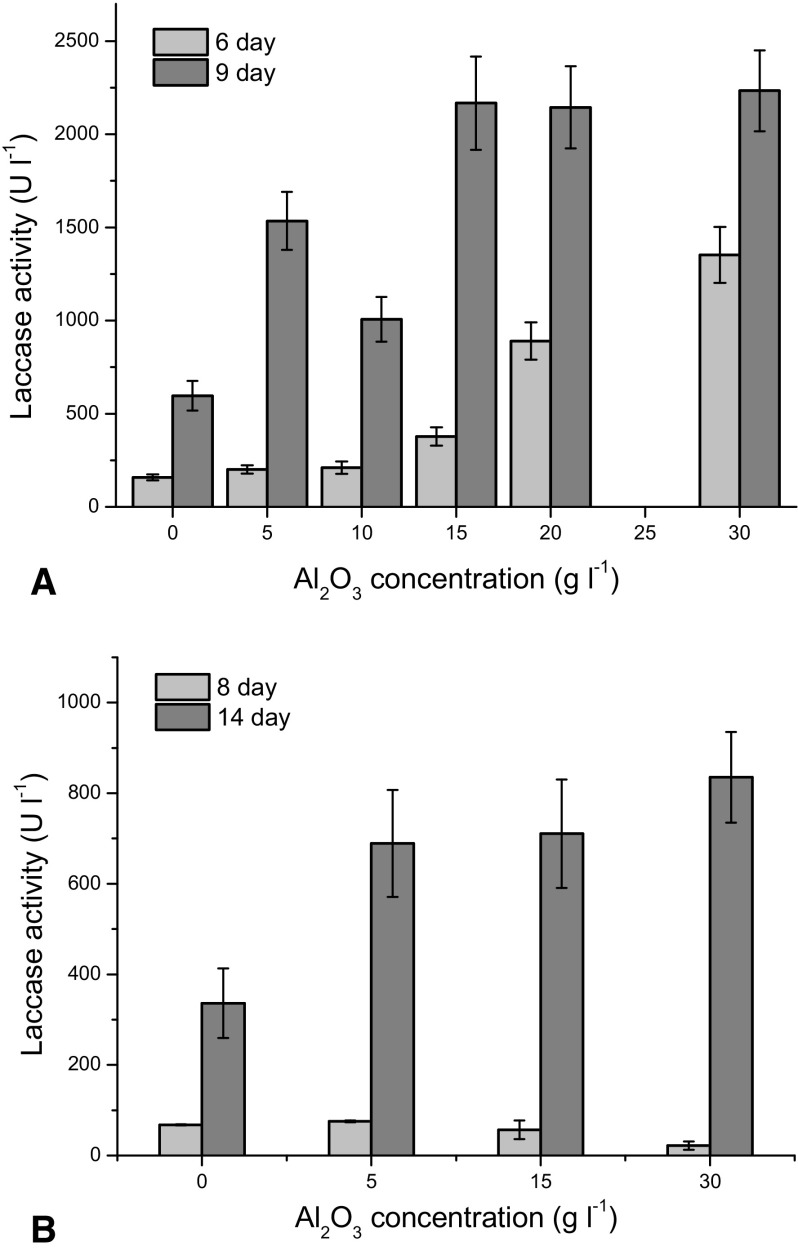
Influence of Al_2_O_3_ microparticles concentration on laccase activity for **a** *C. unicolor* and **b** *P. sapidus*; the presented points represent the averaged values from three experiments and *error bars* are standard deviation

The purpose of the first series of the experiments was to examine the influence of Al_2_O_3_ microparticles on laccase production. For *C. unicolor* laccase activity was detected on 6 day of the cultivation (Fig. 1a) at all tested concentrations of Al_2_O_3_. The positive influence of microparticles on laccase activity was observed starting with 5 g Al_2_O_3_ l^−1^ and next laccase activity increased with the increasing concentration of Al_2_O_3_. On 9 day of the process much higher activities up to 2233 U l^−1^ were observed. At Al_2_O_3_ concentrations equal to 5 and 10 g Al_2_O_3_ l^−1^ 2.5- and 1.7-fold increases of laccase activity were respectively found, while the runs with the addition of 15, 20 and 30 g Al_2_O_3_ l^−1^ resulted in a 3.5-fold increase of laccase activity. The similar effect of the increase of laccase activity was described for *Trametes versicolor* growing on the medium containing CaCO_3_ microparticles (Tišma et al. 2012), in which the several-fold increase of laccase activity was observed. Furthermore, it was found in the current study that increasing microparticle concentrations above 15 g Al_2_O_3_ l^−1^ did not cause any significant change in laccase production by *C. unicolor*. It was similar to the previous findings for *Caldariomyces fumago* culture, in which talc or Al_2_O_3_ concentrations between 10 and 15 g l^−1^ enhanced biomass formation and chloroperoxidase activity but further increase of microparticles concentration had no effect on the culture (Kaup et al. 2008).

A different situation was observed for *P. sapidus* (Fig. 1b) as laccase activity in all runs was not detectable until 8 day of cultivation. There was a 2-days delay compared to *C. unicolor*. But more interesting was the fact that the change of Al_2_O_3_ microparticles concentration did not influence on laccase activity to such an extent as it did for *C. unicolor*. On 14 day of the cultivation laccase activity for *P. sapidus* reached the highest value of 835 U l^−1^ and regardless of Al_2_O_3_ microparticles concentration all laccase activities found were more or less twice higher than those in the control run.

Unlike *P. sapidus*, *C. unicolor*, with regard to microparticles concentration, behaved similarly to the filamentous fungi *A. niger*, whose fructofuranosidase activity changed several-fold with Al_2_O_3_ concentration between 5 and 30 g Al_2_O_3_ l^−1^ (Driouch et al. 2011).

The conducted experiments showed that the mycelial growth of *C. unicolor* in the cultures containing Al_2_O_3_ microparticles, especially at their concentrations exceeding 15 g Al_2_O_3_ l^−1^, was faster. What is more, at Al_2_O_3_ concentrations below 15 g Al_2_O_3_ l^−1^ all microparticles were ultimately built into the mycelial agglomerates (pellets). But at 20 and 30 g Al_2_O_3_ l^−1^ there were microparticles freely suspended in the liquid medium.

In the second series of experiments, Al_2_O_3_ concentrations of 15 and 30 g Al_2_O_3_ l^−1^ were selected for both studied fungal species. Microparticles concentration of 15 g Al_2_O_3_ l^−1^ was believed to be optimal due to the highest laccase activity, visible changes of fungal morphology and the fact that the microparticles were fully built into the fungal agglomerates. Microparticles concentration of 30 g Al_2_O_3_ l^−1^ was chosen because of the high laccase activity on 6 day of the experiment.

**Fig. 2:**
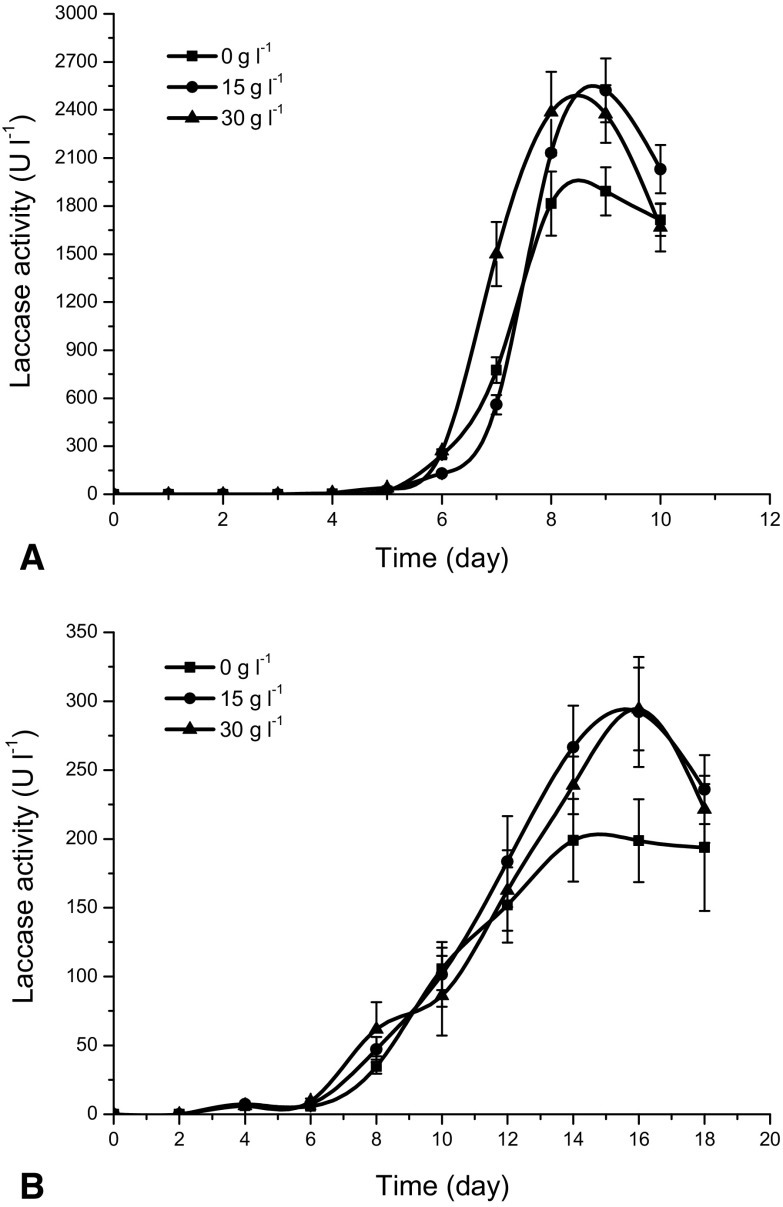
Time changes of laccase activity at various Al_2_O_3_ concentration added to the culture of **a** *C. unicolor* and **b** *P. sapidus*; the presented points represent the averaged values from three experiments and *error bars* are standard deviation

In Fig. 2 it is seen that regardless of Al_2_O_3_ concentration there was no enzymatic activity in the broth before 5 and 6 day of the experiment, respectively for *C. unicolor* (Fig. 2a) and *P. sapidus* (Fig. 2b). For *C. unicolor* at 30 g Al_2_O_3_ l^−1^ the increase in laccase activity was observed on 7 day and was faster than those for the other two experiments. Laccase activity reached then its maximum of 2387 U l^−1^ on 8 day and later began to decrease. For 15 g Al_2_O_3_ l^−1^ a similar increase of laccase activity was observed between 7 and 8 day of the experiment but the maximum activity of 2523 U l^−1^ occurred on 9 day and it was the highest activity out of all experiments.

For *P. sapidus* the highest laccase activity of 294 U l^−1^ was detected on 16 day, i.e. 7 days later than it was for *C. unicolor*. Two tested Al_2_O_3_ concentrations resulted in the similar laccase activities in *P. sapidus*.

**Fig. 3:**
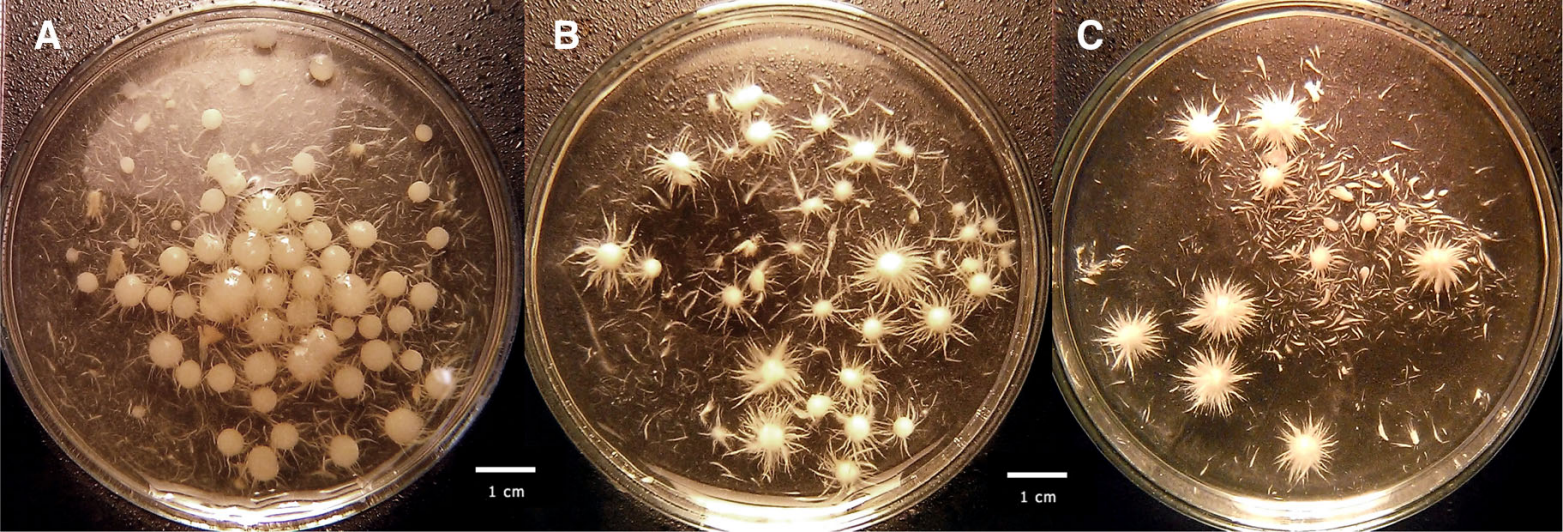
Influence of Al_2_O_3_ microparticles added to the culture medium on *C. unicolor* morphology: **a** control run without microparticles, **b** 15 g Al_2_O_3_ l^−1^ and **c** 30 g Al_2_O_3_ l^−1^; *scale-bar* size 1 cm

**Fig. 4:**
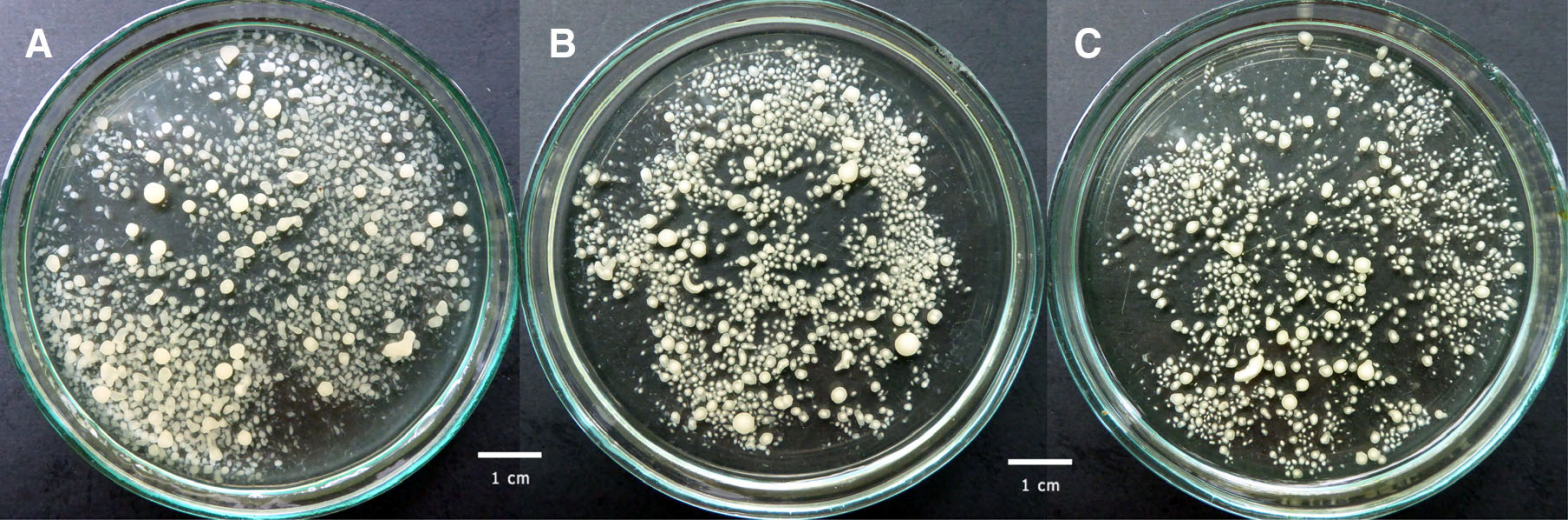
Influence of Al_2_O_3_ microparticles added to the culture medium on *P. sapidus* morphology: **a** control run without microparticles, **b** 15 g Al_2_O_3_ l^−1^ and **c** 30 g Al_2_O_3_ l^−1^; *scale-bar* size 1 cm

All these changes in laccase activity for both studied species were attributed to the changes in the size and shape of agglomerates (pellets), namely fungal morphology. The images of growing biomass are presented in Fig. 3 for *C. unicolor* and in Fig. 4 for *P. sapidus*. For both fungal strains the change in the size and shape as well as in the structure of the pellets were observed.

For *C. unicolor* when no microparticles were added (Fig. 3a) smooth, sometimes slightly fluffy pellets with some dispersed hyphae were observed. Diameters of pellets ranged from 2 to 6 mm and were significantly higher than those of pellets coming from the media containing Al_2_O_3_ microparticles. In the case of 15 g Al_2_O_3_ l^−1^ added (Fig. 3b) hairy and star-shaped pellets were formed. Their diameters were lower than those in the run without microparticles and ranged from 1 to 4 mm. Biomass from the cultures, to which 30 g Al_2_O_3_ l^−1^ were added (Fig. 3c), grew partly in the form of pellets but also much dispersed hyphae were observed. Also these pellets had a characteristic star-shaped form and looser structure. The size of the pellets was between 1 and 3 mm. The similar decrease in the pellet size and formation of freely dispersed mycelium was also observed by Driouch et al. (2011) for *A. niger* morphologically engineered with the use of either talc or Al_2_O_3_ microparticles.

For *P. sapidus* (Fig. 4a–c) the addition of microparticles led to the formation of smaller but more compact pellets. However, no significant difference in pellet morphology at various Al_2_O_3_ concentrations was seen. In many cases there was a visible core in the centre of the pellet, in which the microparticles were incorporated (Fig. 4b, c). This type of pellets was previously called core–shell pellets by Driouch et al. (2012) and these authors obtained them with the use of titanate microparticles towards fructofuranosidase and glucoamylase producer *A. niger*. Opposite to *C. unicolor* cultivations there were no remaining microparticles in the medium even at 30 g Al_2_O_3_ l^−1^. It meant that all added microparticles were built into *P. sapidus* biomass.

The formation of smaller pellets exerted by the addition of microparticles is the phenomenon often described in literature for *Aspergilli* (Walisko et al. 2012). It is actually the aim of microparticle-enhanced cultivation to produce smaller and less dense pellets by interfering with agglomeration of fungal spores. Here, more interesting is the fact that the microparticles acted directly on the agglomeration of free hyphae and the change in structure of the fungal morphology was different for two basidiomycetes strains studied.

Summing up, as the increase of laccase activity occurred to be significant and the microparticles used are easily available and inexpensive, MPEC is a simple and effective strategy to intensify biotechnological laccase production by basidiomycetes.

## Conclusions

1. The applied Al_2_O_3_ microparticles in the culture of two basidiomycetes species at concentration 15 g Al_2_O_3_ l^−1^ cause the increase of laccase activities, respectively 3.5-fold for *C. unicolor* and 2-fold for *P. sapidus* compared to the control run without microparticles.
2. Addition of Al_2_O_3_ microparticles decreases pellet size for both studied species but the changes in the structure of agglomerates are different for each of them. *C. unicolor* pellets get more hairy up to the transformation into dispersed morphology. For *P. sapidus* pellets are more compact and have the characteristic core with the incorporated microparticles.
